# Unlocking the potential of exosomes: a breakthrough in the theranosis of degenerative orthopaedic diseases

**DOI:** 10.3389/fbioe.2024.1377142

**Published:** 2024-04-18

**Authors:** Yaohang Yue, Wei Dai, Yihao Wei, Siyang Cao, Shuai Liao, Aikang Li, Peng Liu, Jianjing Lin, Hui Zeng

**Affiliations:** ^1^ School of Clinical Medicine, Shandong Second Medical University, Weifang, Shandong, China; ^2^ Peking University Shenzhen Hospital, Shenzhen, Guangdong, China; ^3^ Department of Bone and Joint Surgery, Peking University Shenzhen Hospital, Shenzhen, Guangdong, China; ^4^ National and Local Joint Engineering Research Centre of Orthopaedic Biomaterials, Peking University Shenzhen Hospital, Shenzhen, Guangdong, China; ^5^ Shenzhen Key Laboratory of Orthopaedic Diseases and Biomaterials Research, Peking University Shenzhen Hospital, Shenzhen, Guangdong, China; ^6^ Department of Sports Medicine and Rehabilitation, Peking University Shenzhen Hospital, Shenzhen, Guangdong, China; ^7^ Shenzhen Second People’s Hospital, Shenzhen, Guangdong, China

**Keywords:** exosomes, osteoarthritis, osteoporosis, intervertebral disc degeneration, treatment

## Abstract

Degenerative orthopaedic diseases pose a notable worldwide public health issue attributable to the global aging population. Conventional medical approaches, encompassing physical therapy, pharmaceutical interventions, and surgical methods, face obstacles in halting or reversing the degenerative process. In recent times, exosome-based therapy has gained widespread acceptance and popularity as an effective treatment for degenerative orthopaedic diseases. This therapeutic approach holds the potential for “cell-free” tissue regeneration. Exosomes, membranous vesicles resulting from the fusion of intracellular multivesicles with the cell membrane, are released into the extracellular matrix. Addressing challenges such as the rapid elimination of natural exosomes *in vivo* and the limitation of drug concentration can be effectively achieved through various strategies, including engineering modification, gene overexpression modification, and biomaterial binding. This review provides a concise overview of the source, classification, and preparation methods of exosomes, followed by an in-depth analysis of their functions and potential applications. Furthermore, the review explores various strategies for utilizing exosomes in the treatment of degenerative orthopaedic diseases, encompassing engineering modification, gene overexpression, and biomaterial binding. The primary objective is to provide a fresh viewpoint on the utilization of exosomes in addressing bone degenerative conditions and to support the practical application of exosomes in the theranosis of degenerative orthopaedic diseases.

## 1 Introduction

Exosomes, membrane-bound nanovesicles, encompass a diverse array of biomolecules, including lipids, proteins, and nucleic acids. These nanovesicles are secreted by cells through exocytosis, a process facilitating their uptake by target cells, thereby enabling the transmission of biological signals between local or distant cells (He et al., 2018). Under both physiological and pathological conditions, nearly all cell types demonstrate the capability to release exosomes (Ludwig and Giebel, 2012). Moreover, exosomes assume a pivotal role in cellular communication, mediating the transfer of nucleic acids and specific proteins and lipids between cells. This function holds particular significance in maintaining protein and lipid homeostasis (Christianson et al., 2013).

Exosomes hold a pivotal position in a range of physiological processes and pathological reactions, encompassing neuronal pathways (Men et al., 2019), antigen presentation (Lindenbergh and Stoorvogel, 2018), organ development (Melnik et al., 2021), immune response (Xu et al., 2020), inflammation (Noonin and Thongboonkerd, 2021), cancer progression (Zhang and Yu, 2019), as well as cardiovascular and cerebrovascular diseases (Han et al., 2022) and neurodegenerative diseases (Hill, 2019). Within the domain of orthopedic degenerative disorders, exosomes also play a substantial role in conditions like osteoarthritis (OA), osteoporosis (OP), intervertebral disc degeneration (IDD), and other similar ailments within the field of orthopedic degenerative disorders. The global aging population has elevated degenerative orthopaedic diseases to a pressing global public health concern. Notably, OA, OP, and IDD stand out as the most prevalent degenerative orthopaedic diseases, inflicting pain, disability, and imposing a substantial social burden within the context of an aging populace (Hopkins and Chen, 2020; Chen et al., 2022). In spite of the enduring utilization of traditional medical approaches, such as physical therapy, pharmacological intervention, and surgical procedures, aimed at impeding or reversing the degenerative process, their effectiveness remains constrained in altering the progression of degenerative diseases. (Wu et al., 2022). The bone, osteochondral, and cartilage regions, extensively utilized in the human body, exhibit constrained regenerative capacity due to compromised vasculature in severe defects and the avascular nature of cartilage. Consequently, these tissues struggle to fully recover from injuries, diseases, or wear, hampered by inadequate nutrient supply and cellular support (Chen et al., 2020a; Chen et al., 2021).

Various stressors can cause chronic inflammation, leading to the release of inflammatory substances such as interleukin-6 (IL-6), Interleukin-1 (IL-1), tumor necrosis factor-α (TNF-α), and prostaglandin E2 (PGE2). This cascade of inflammatory responses can disrupt the delicate equilibrium between osteoblasts and osteoclasts, resulting in metabolic disorders within the skeletal system (Alippe and Mbalaviele, 2019). Additionally, these inflammatory factors possess the potential to adversely affect chondrocytes, initiating the degradation of the cartilage matrix (Henrotin et al., 1996). Recognizing exosomes as transmitters of intercellular information opens avenues for exploring their role in the pathogenesis and therapeutic targets of orthopaedic diseases.

Exosome have garnered significant attention as a subject of study in various medical fields, including cancer, cardiovascular diseases, and neurological disorders (Cheng and Hill, 2022). However, existing literature on the involvement of exosome in bone degenerative diseases has primarily concentrated on therapeutic interventions. This review aims to present a comprehensive analysis of the role of exosome in bone degenerative diseases, with a particular focus on diagnostic and therapeutic strategies, as well as an examination of pertinent obstacles and constraints. The aim of this study is to provide a comprehensive summary of various critical elements, including (He et al., 2018): an examination of the origins and isolation techniques of exosome (Ludwig and Giebel, 2012); an analysis of the biological distribution of exosome, their potential for drug delivery, and strategies for modification (Christianson et al., 2013); an exploration of the diagnostic utility of exosome in bone degenerative diseases, as well as the therapeutic efficacy of both modified and unmodified exosome in treating these conditions, and their potential as carriers for controlled release of bioactive substances. Furthermore, the future prospects for the advancement of exosome in the realm of bone degenerative diseases are also considered.

## 2 Summary of exosomes

Exosomes, nanoscale membrane vesicles resulting from the fusion of organelles in the endocytic pathway with the plasma membrane, have garnered significant research attention in recent years. This interest is attributed to their crucial role in health and disease, as well as their potential clinical applications in treatment and diagnosis (Hessvik and Llorente, 2018). As a category of extracellular vesicles (EVs) discharged by all cells, encompassing both prokaryotes and eukaryotes, exosomes have surfaced as innovative agents in the communication between cells in various health and disease contexts. Intriguingly, even though they were initially perceived as unwanted cellular discards when first identified 3 decades ago, exosomes are presently acknowledged as conveyors that expedite the conveyance of external substances—such as proteins, mRNAs, microRNAs, and lipids—from donor cells to recipient cells (Johnstone et al., 1987; Deatherage and Cookson, 2012).

Exosomes have a crucial function in numerous physiological and pathological processes. They aid in the transmission of nucleic acids, proteins, or lipids to target cells, enabling communication between different cells. The transfer of this induces alterations in the phenotype and function of the cells being targeted (Wortzel et al., 2019). Exosomes have garnered significant attention for their potential as diagnostic biomarkers and vehicles for therapy, leading to a growing interest in their clinical applications. Their biocompatibility and bilayer lipid structure contribute to reduced immunogenicity, thereby protecting genetic material from degradation and rendering them attractive for therapeutic purposes. Furthermore, their petite dimensions and membrane constitution empower them to traverse prominent biofilms, encompassing the blood-brain barrier (Haney et al., 2015; Zheng et al., 2019).

Exosomes have proven to be useful in the transportation of therapeutic drugs, acting as carriers for medications like small molecules or nucleic acid drugs (Batrakova and Kim, 2015). This approach enables the incorporation of drugs into exosomes, facilitating targeted delivery to specific cell types or tissues. The potential of targeted drug delivery is to increase the concentration of therapeutic drugs in a specific area while reducing the occurrence of side effects (Liang et al., 2021). Nevertheless, the introduction of inherent exosomes is accompanied by disadvantages, such as swift dispersion, degradation, and variability in functional content, curtailing their effectiveness in intricate healing procedures. In order to more aptly meet the demands of the regeneration process, diverse improved approaches for exosomes have been devised, including modifications through engineering, overexpression of genes, and the delivery of biomaterials.

## 3 Origin of exosomes

**FIGURE 1:**
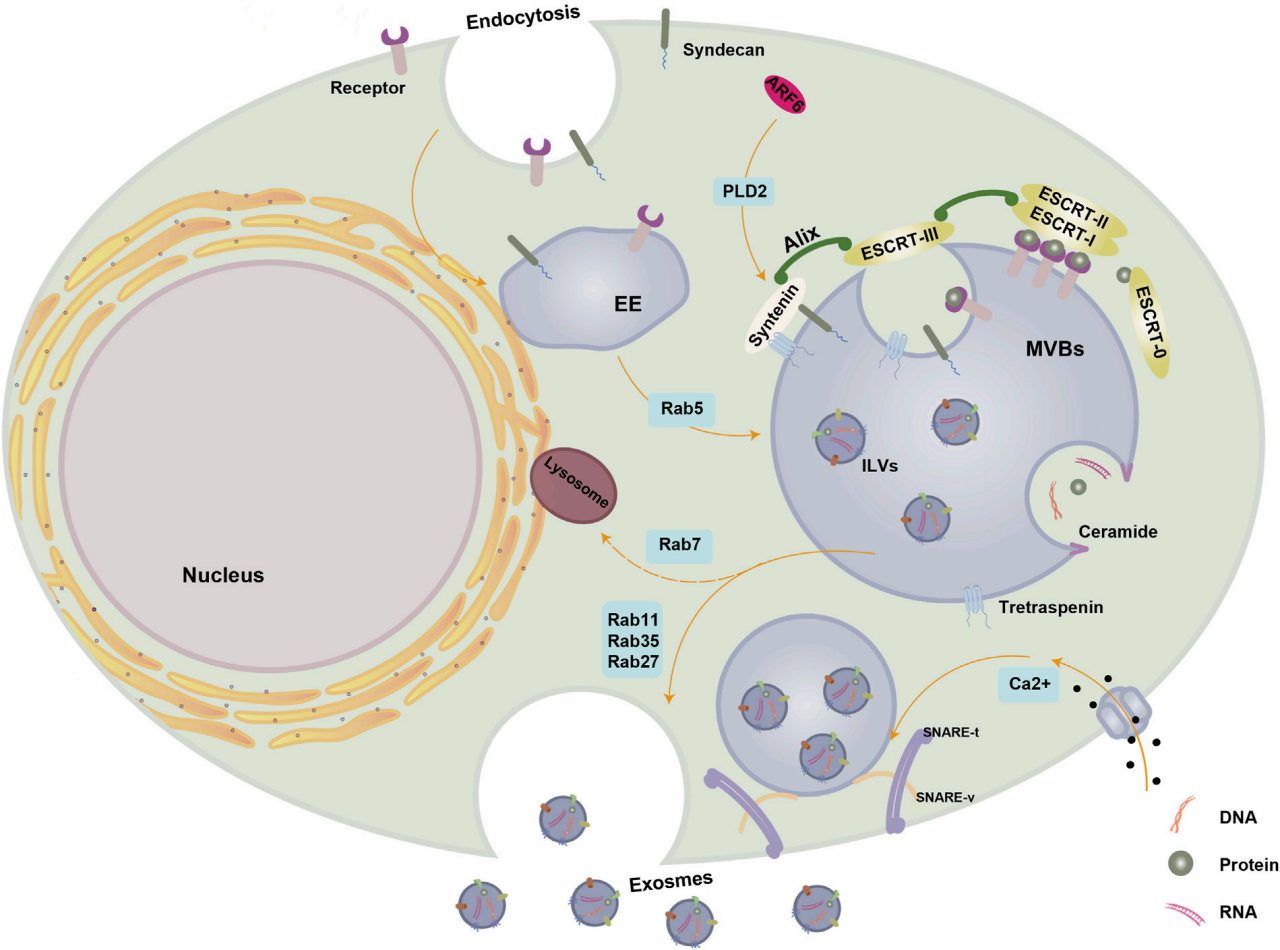
Biogenesis of exosomes. Exosome biogenesis and secretion are regulated by a variety of molecules. The small GTPase Rab5 facilitates the maturation of EE. Within MVBs, cytoplasmic cargoes undergo segregation within ILVs through both ESCRT-dependent and ESCRT-independent mechanisms. The ALIX-syntenin-syndecan pathway also plays a role in MVB maturation and is under the control of ARF6 and its effector PLD2. Ceramide induces the inward budding of MVBs limiting membranes, leading to ILVs formation. Rab11, Rab35, and Rab27 contribute to MVBs maturation, transportation, and docking, while Rab7 promotes the formation of MVBs destined for degradation through fusion with lysosomes. SNARE and cytoskeletal proteins facilitate membrane remodeling and drive the fusion of MVBs with the PM for exosome release, with Ca2+ essential for SNARE complex assembly. Abbreviations: PM, plasma membrane; EE, early endosome; ILVs, intraluminal vesicles; MVBs, multivesicular bodies; ER, endoplasmic reticulum; ESCRT, endosomal sorting complex required for transport; SNARE, soluble N-ethylmaleimide-sensitive fusion attachment protein receptor; ALIX, apoptosis-linked gene two-interacting protein X; ARF6, ADP ribosylation factor 6; PLD2, phospholipase D2. This figure was created using Figdraw (https://www.figdraw.com/static/index.html#/).

Exosomes originate from the endosomal system, where early endosomes progress into late endosomes or multivesicular bodies (MVBs). During this maturation, the endosomal membrane undergoes invagination, leading to the generation of intraluminal vesicles (ILVs) within the organelle’s lumen (Huotari and Helenius, 2011). MVBs and late endosomes are rich in ILVs, allowing the sequestration of specific proteins, lipids, and cytosolic components. ILVs result from the inward budding of endosomal membranes and transform into exosomes, a phenomenon initially observed in the secretion of transferrin receptor (TFR) vesicles from mature reticulocytes (Pan and Johnstone, 1983). As part of extracellular vesicles (EVs), exosomes constitute the ILV of MVBs, typically measuring 30–100 nm in diameter (Camussi et al., 2011; György et al., 2011) (Figure 1). ILVs are mainly produced and released through the endosomal sorting complex necessary for transportation (ESCRT) mechanism, which is a crucial intracellular multi-subunit system responsible for modifying membranes. ESCRT, which consists of four separate protein complexes (ESCRT-0, ESCRT-I, ESCRT-II, ESCRT-III) and the associated AAA ATPase Vps4 complex, plays a role in the formation of vesicle buds and the organization of cargo in MVBs (Henne et al., 2013). The ESCRT-0 complex identifies and isolates proteins that have been ubiquitinated in the membrane of endosomes, whereas ESCRT-I and -II complexes aid in altering the membrane by creating buds that contain the isolated cargo. Following that, ESCRT-III constituents facilitate the division of vesicles (Hurley and Hanson, 2010).

The ESCRT-0 complex is composed of HRS, a protein that identifies monoubiquitinated cargo proteins, and interacts with STAM, eps15, and clathrin to form a complex involved in signal transduction. HRS enlists TSG101 from the ESCRT-I complex, which then involves ESCRT-I in enlisting ESCRT-III via ESCRT-II or Alix (, apoptosis-linked gene two-interacting protein X). In the end, the separation and reusing of the ESCRT process require engagement with the Vps4 AAA ATPase. While ESCRT-independent mechanisms contribute to MVB/exosome biogenesis, the formation and secretion of exosome populations also rely on the functions of ESCRT and accessory proteins. Protein sorting and the production of ILVs that are later released as exosomes involve only specific components (Colombo et al., 2013).

The Alix-syntenin-syndecan pathway also participates in cargo sorting during MVB formation. Together with ESCRT-III components, the syndecan-syntenin-Alix complex aids in cargo segregation and ILV inward budding (Juan and Fürthauer, 2018). ADP ribosylation factor 6 (ARF6) and its effector, phospholipase D2 (PLD2), regulate this pathway (Ghossoub et al., 2014).

According to recent research, the formation of MVBs can happen without the involvement of ESCRT. For example, even if the primary components of all four ESCRT complexes are muted at the same time, ILVs continue to develop inside MVBs. This observation underscores the existence of mechanisms for MVB formation that operate independently of ESCRT (Stuffers et al., 2009).

Exosomes contain ceramide, which triggers the first found mechanism of exosome formation independent of ESCRT (Trajkovic et al., 2008). A negatively curved membrane may result from ceramide’s cone-shaped structure. Rab GTPases play a pivotal role as membrane-associated proteins involved in nearly all intracellular vesicle trafficking processes. As MVBs mature, transport, and dock, Rab11, Rab35, and Rab27 play a crucial role, while Rab7 contributes to their formation. (Schwartz et al., 2007). In other study, Wei et al. highlighted the significance of Rab-GTPase Rab31 in regulating exosome biogenesis. Rab31 promotes the generation of ILVs without relying on the typical ESCRT pathway and hinders the merging of MVBs with lysosomes. Furthermore, Rab31 serves as a marker and controller of the ESCRT-independent exosome pathway (Wei et al., 2021). In addition to Rab227a/b, another Rab GTPase is responsible for regulating the fusion of MVBs with the plasma membrane to release exosomes (Ostrowski et al., 2010). Phosphorylation by the epidermal growth factor receptor (EGFR) is essential for the activation of Rab31, which is pivotal in the transportation of proteins within lipid raft microdomains. This drives EGFR into the multivesicular endosome (MVE), forming ILVs independently of the ESCRT mechanism. Rab31 interacts with the Stomainh, prohibitin, Flotillin, and Hflk/C (SPFH) domain, driving ILV formation through the flotillin domain of Flutirin. At the same time, Rab31 enlists the GTPase deactivating protein Tbc1d2b to deactivate Rab7, which stops the merging of MVE with lysosomes and aids in the release of ILV as exosomes (Wei et al., 2021).

Lipids have a vital function in the transportation of vesicles, and it has been confirmed that the proteolipid protein (PLP) is transferred to ILVs from the lipid-abundant section of endosomal membranes. Significantly, this procedure takes place autonomously from the ESCRT. Cholesterol, ceramide, and sphingomyelin are among the components found in the lipid-rich section. The ceramide-rich region of endosomes has demonstrated high sensitivity to inward budding, and any defects in sphingomyelinase—leading to the conversion of sphingomyelin to ceramide—inhibit ILV formation (McGough and Vincent, 2016).

Furthermore, as transmembrane proteins and highly conserved membrane integral proteins abundant in exosomes, tetraspanins play a crucial role in ESCRT-independent exosome release. They aggregate membrane-associated molecules in regions prone to intraluminal vesicle (ILV) formation and contribute to protein scaffolding and anchoring in cell membranes (Escola et al., 1998; Hemler, 2003). Notably, tetraspanins CD9, CD63, and CD81 are prevalent in exosomes and are commonly utilized as biomarkers for exosomes. Studies have demonstrated that the expression of tetraspanins CD9 and CD81 actively promotes the release of exosomes (Chairoungdua et al., 2010).

While the biogenesis of exosomes is typically characterized as either ESCRT-dependent or ESCRT-independent mechanisms, it is important to note that these pathways may not be entirely distinct (Maas et al., 2017).

## 4 Classification and isolation methods of exosomes

### 4.1 Classification of exosomes

Exosomes can be categorized into natural exosomes and engineered exosomes based on whether they have undergone artificial modifications. Natural exosomes, in turn, are further subdivided into animal-derived exosomes and plant-derived exosomes. Given that exosomes are generated under both normal and tumor conditions, animal-derived exosomes can be specifically classified as normal exosomes and tumor exosomes (Zhang et al., 2020a).

### 4.2 Isolation method of exosomes

Exosome isolation methods commonly used include Ultracentrifugation (UC), polymer precipitation, size-based techniques, and Immunoaffinity capture chromatography (ICC) (Table1).

**TABLE 1:** Advantages and disadvantages of extracting exosomes.

Method	Principle	Advantage	Disadvantage	Reference
ultracentrifugation	Separation based on differences in size and density of each component in the original solution	No need to mark exosome to avoid cross contamination	Time consuming, high cost, structural damage, and agglomeration	Ludwig and Giebel (2012), Christianson et al. (2013), He et al. (2018), Lindenbergh and Stoorvogel (2018), Men et al. (2019), Xu et al. (2020), Melnik et al. (2021)
density gradient centrifugation	Separation using differences in size and density between exosomes and impurity particles	Better separation of exosome and improvement of its purity	Reduce the sedimentation rate of exosome, taking longer time	Hill, 2019; Zhang and Yu (2019), Noonin and Thongboonkerd (2021), Han et al. (2022)
Polymer precipitation	Harvesting exosome under centrifugal conditions by reducing the solubility of exosome	Simple and fast, suitable for handling large doses of samples	Low purity and recovery rate, resulting in false positives, making it difficult to remove the resulting polymer	Hopkins and Chen (2020), Chen et al. (2022), Wu et al. (2022)
ultrafiltration	Using pressure and flow rate, asymmetric microporous structure and semi-permeable membrane allow solvent and small molecules to pass through in cross-flow filtration	high speed, quantity, and particle size	low recovery rate and purity	Chen et al. (2020a), Chen et al. (2021)
size exclusion chromatography	Separation is carried out by filling the resin in the chromatographic column based on differences in molecular size	Fast, simple, and low-cost application	Excessive labor, sample contamination, and protein aggregation	Alippe and Mbalaviele (2019)
Immunoaffinity capture chromatography	Specific binding based on antibodies and ligands	Strong specificity, high sensitivity, high purity, and high yield	Storage conditions are harsh, not suitable for large-scale manufacturing, and can produce interfering proteins	Henrotin et al. (1996), Melnik et al. (2021), Cheng and Hill (2022)

#### 4.2.1 UC

Ultracentrifugation (UC) stands as the predominant technology and gold standard for extracting and isolating exosomes. By utilizing variations in size and density, this technique carefully isolates crucial components from a solution. Traditional UC is commonly used in processing biofluids such as urine, serum, aqueous humor, cerebrospinal fluid, amniotic fluid, and breast milk (Helwa et al., 2017). UC is highly efficient in segregating sample components with substantial differences in sedimentation coefficient (Livshits et al., 2015). The initial application of UC for exosome isolation was pioneered by Johnstone et al.in the extraction of exosomes from reticulocyte tissue culture medium (Johnstone, 1992). The UC isolation process involves two main steps. Initially, a sequence of consecutive centrifugation steps at low to moderate speeds is employed to remove dead cells, cellular waste, and sizable EVs. Afterwards, exosomes are separated more quickly by applying a centrifugal force of 100,000 g and then cleansed with PBS to eliminate impurities, including unwanted proteins. By employing this method, the requirement for exosome labeling is eradicated, and the potential for cross-contamination is greatly reduced. A number of factors, including the type of rotor, the centrifugation time, and the viscosity of the sample, impact the yield and purity of exosomes obtained using UC. (Cvjetkovic et al., 2014; Jeppesen et al., 2014). UC protocols that are employed and optimized for specific sample types must consider these parameters. Nonetheless, downstream analysis of it encounters challenges attributed to its time-intensive procedures, elevated cost, potential structural impairments, aggregation issues, and co-separation with lipoproteins. (Böing et al., 2014; Yang et al., 2019a).

Some studies have suggested that repeated ultracentrifugation (UC) results in reduced exosome yields and detrimental effects on exosome quality, rendering them unsuitable for clinical applications. Furthermore, high-speed centrifugation may cause damaged exosomes due to the high shear forces (Mol et al., 2017; Konoshenko et al., 2018).

#### 4.2.2 Density gradient centrifugation

To enhance exosome purity, density gradient centrifugation is commonly combined with UC. Density gradient centrifugation is a more advanced version of traditional differential ultracentrifugation, using inert centrifugal medium like sucrose and iodixanol to separate exosomes based on density. Sucrose density gradient UC, as a widely used method, utilizes UC and sucrose density gradient to separate exosomes, providing an advantage in the isolation of organelles, proteins, and other biomolecules. While this method improves the purity of exosomes, the higher thickness of the sucrose solution slows down the settling speed of exosomes, consequently prolonging the extraction procedure (Ford et al., 1994; Chen et al., 2019). In contrast, the iodixanol density gradient centrifugation method presents numerous advantages over the sucrose density gradient centrifugation method, such as decreased viscosity, metabolic inertness, non-toxicity to cells, and enhanced preservation of cell integrity and functionality. Konadu et al. separated exosomes from HIV-1 particles in infected individuals’ plasma using iodixanol velocity gradients. Exosomes were found in upper fractions with lower density, while viral particles were in lower fractions with higher density (Konadu et al., 2016).

Density gradient centrifugation improves exosome isolation purity compared to ultracentrifugation, but it is complex, requires high technical skill, has low throughput, is time-consuming, and does not effectively remove lipoproteins and chylomicrons from blood samples (Wang et al., 2024a).

#### 4.2.3 Polymer precipitation

Precipitation techniques use polymers like polyethylene glycol (PEG) to isolate and purify exosomes, with PEG being the most commonly used polymer for this purpose. (Oh et al., 1988). PEG modifies the cell membrane surface structure, decreasing the degradation of endogenous lipids and phospholipids in the sediment. Exosomes aggregate due to cell adhesion and surface tension, forming aggregates that precipitate with PEG (Prabhu and Balasubramanian, 2001). Simple, fast, minimally damaging exosomes, and low equipment requirements make precipitation methods attractive for clinical research. Nevertheless, the level of purity and the rate of recovery are comparatively inadequate, which may result in inaccurate positive outcomes. Additionally, the resulting polymer is challenging to remove, hindering subsequent functional experimental analysis. This challenge can be overcome by adding an extra step of purification or filtration (Afridi et al., 2023).

Contemporary precipitation techniques are advantageous for clinical use due to their requirement of minimal starting material for the manipulation of human biofluids and their compatibility with high-throughput capabilities.

#### 4.2.4 Size-based isolation techniques

This technology primarily focuses on separating exosomes and other elements in biological samples based on their sizes using ultrafiltration and size exclusion chromatography (SEC).

Exosomes are isolated from large volumes of biofluids using ultrafine nanomembranes with different molecular weight cutoffs in ultrafiltration. The separation is based on exosome size and includes three steps: dead-end filtration, tangential flow filtration, and track-etched membrane filtration. (Li et al., 2017). Researchers compared UC and ultrafiltration for isolating exosomes to diagnose lung cancer. Ultrafiltration showed advantages in speed, quantity, and particle size (<100 nm) (Lobb et al., 2015). However, this method has limitations, such as a low recovery rate and purity. Moreover, there is a potential risk of filters becoming clogged with external debris (Li et al., 2017).

Compared to centrifugal and filtration methods, SEC offers several advantages, including cost-effectiveness, non-destructive outcomes, and reproducibility. The underlying principle of SEC is the inability of macromolecules to penetrate gel pores, leading to their elution with the mobile phase through the interstitial spaces between the porous gels. In contrast, smaller molecules are retained within the gel pores and subsequently elute with the mobile phase. Drawbacks associated with this methodology include substantial labor requirements, potential sample contamination with lipoproteins, and the risk of protein aggregation (Witwer et al., 2013).

Although size-based techniques are widely used in various fields, they often have long running times, limiting their usefulness in treatment and research.

#### 4.2.5 ICC

ICC utilizes exosome surface biomarkers to capture exosomes by binding antibodies to magnetic beads, plates, chromatographic matrices, or microfluidic devices, forming covalent bonds. These biomarkers specifically bind to antigens or membrane proteins on the exosomes (Yang et al., 2019a; Zhang et al., 2020a). Exosomes are measured in plasma, serum, and urine using ICC-based techniques such as ELISA, magneto-immunoprecipitation, and Western blotting (Mahgoub et al., 2020). Immunoaffinity chromatography isolates and purifies substances from complex mixtures using antibodies and ligands, resulting in strong specificity, exceptional purity, increased sensitivity, and significant yield. The strict storage needs for ICC-obtained exosomes make large-scale isolation impractical due to possible interference adsorption.

In general, this method is appropriate for both qualitative and quantitative analysis of exosomes. Nevertheless, various limitations, including its elevated cost, limited yield, stringent handling protocols, and specific storage prerequisites, restrict its widespread utility (Zhang et al., 2020a).

## 5 Biological distribution and drug delivery of exosomes

### 5.1 Biological distribution

Exosomes facilitate both local and systemic intercellular communication and are released by various cell populations, including dendritic cells, macrophages, cancer cells, and mesenchymal stem cells (Morishita et al., 2017). These exosomes can be found in diverse bodily fluids such as breast milk, blood, serum, urine, saliva, amniotic fluid, and synovial fluid (Qin and Xu, 2014). Moreover, exosomes may undergo multiple cycles of cellular uptake and release before entering multilayered tissues (Lakhal and Wood, 2011). Studies involving exosomes obtained from cells and bodily fluids, such as those derived from milk, reveal that upon oral consumption, they distribute throughout different organs, encompassing the liver, lungs, kidneys, pancreas, spleen, ovaries, colon, and brain. On the other hand, when administered intravenously, the liver is mainly affected, followed by the spleen, lung, and gastrointestinal tract (Munagala et al., 2016; Morishita et al., 2017; Murphy et al., 2019). Swift elimination from the bloodstream occurs with intravenous exosome injection, whereas intra-tumoral injection prolongs tumor exosome detection, mainly because circulating phagocytes like macrophages and neutrophils are rapidly eliminated (Smyth et al., 2015). The retention time of exosomes in tissues like the liver and spleen significantly exceeds that in blood, persisting for over 24 h (Faruqu et al., 2019).

### 5.2 Drug delivery

Various methods can be used to utilize exosomes as carriers in drug delivery, including incorporating drugs into exosomes from naive parental cells, loading drugs into parental cells and releasing them through exosomes, and transfecting or infecting parental cells with DNA that encodes therapeutic compounds released through exosomes. The choice of method depends on factors such as the specific drug, disease site, and conditions conducive to delivery. (Batrakova and Kim, 2015). Numerous methods have been suggested for the *in vitro* loading of inexperienced exosomes, wherein lipophilic compounds of small size are commonly loaded passively by co-incubating with exosomes or vesicles resembling exosomes. In addition, EVs, like exosomes, naturally carry a wide variety of biomolecules such as messenger RNAs, microRNAs, non-coding RNAs, mitochondrial DNA, and genomic DNA (Théry et al., 2009; Waldenström et al., 2012). As a result, exosomes are regarded as promising carriers for transferring nucleic acid, and electroporation of purified exosomes has been proposed as a means of loading exogenous RNA into living cells (Alvarez-Erviti et al., 2011; El Andaloussi et al., 2013; Lai et al., 2013).

Alvarez Erviti et al. first developed the method of introducing siRNA into exosomes obtained from dendritic cells through electroporation (Alvarez-Erviti et al., 2011). The same approach was employed to introduce miRNAs into breast cancer cells expressing EGFR (Ohno et al., 2013). In an alternative method, exogenous compounds can be introduced into parental cells and subsequently released into the conditioned medium within exosomes. For instance, exosomes derived from mesenchymal stem cells were infused with paclitaxel by incubating the original cells with the medication (Pascucci et al., 2014). Introducing ovac1c2 fusion complementary DNA (cDNA) into cells caused chicken egg ovalbumin to be incorporated into vesicles in the cell membrane (Zeelenberg et al., 2008). This method helped develop a new drug delivery system for treating neurodegenerative diseases by exposing macrophages to plasmid DNA (pDNA) with therapeutic proteins such as catalase or glial cell line-derived neurotrophic factor (GDNF). (Haney et al., 2013; Zhao et al., 2014).

## 6 Improvement strategies for exosomes

Exosomes, which are nanovesicles derived from cells, have a vital function in promoting the intercellular transfer of different substances. By encapsulating therapeutic agents such as small molecules or nucleic acid drugs, exosomes can serve as vehicles for targeted delivery to specific cell types or tissues. This approach has the potential to enhance the localized concentration of therapeutic drugs while simultaneously mitigating undesirable side effects (Liang et al., 2021).

Historically, exosomes were derived from various cellular sources and directly administered into degenerative sites in their unaltered form. However, the use of unmodified exosomes had certain limitations, including swift clearance and excessively rapid release, hindering their effectiveness in complex healing procedures. To better meet the demands of disease management, researchers have developed several enhanced exosome strategies. These include engineered surface modificationsand biomaterial delivery, aiming to facilitate more accurate targeting and treatment approaches.

### 6.1 Engineering Surface modification

**FIGURE 2:**
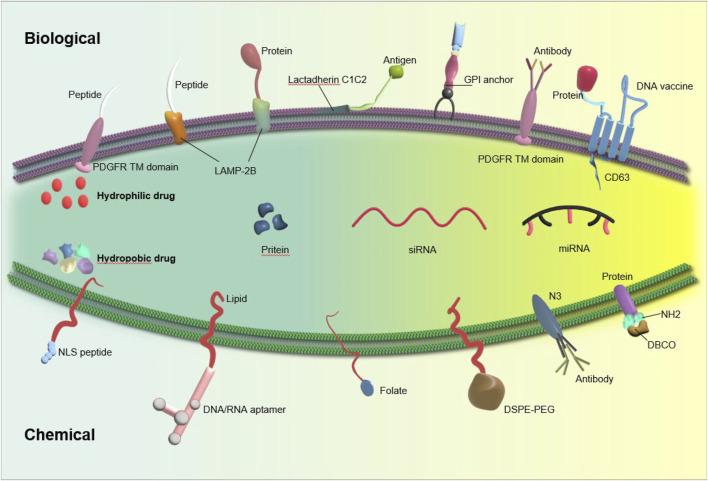
Exosome stability can be enhanced through exosome engineering techniques such as physical or chemical treatment and surface modification, leading to improved delivery efficiency.

Although exosomes originate naturally, their surface can be readily altered. The main goal of surface modification engineering is to provide targeting specificity to particular cell types using genetic engineering and chemical modification techniques (Alvarez-Erviti et al., 2011) (Figure 2).

#### 6.1.1 Genetic manipulation

The membrane of the exosome consists of transmembrane proteins such as Lamp, GPI, and tetraspanins (such as CD63, CD9, CD81), which have the potential to fuse with targeting ligands, thereby improving the accuracy of exosome delivery (Mentkowski et al., 2018). One common method for generating surface-modified exosomes (SMEs) involves using plasmid vectors to genetically modify cells that produce exosomes. This modification entails fusing a targeting ligand with one of the aforementioned transmembrane proteins.

Lamp2b is often used to modify exosomal surface proteins for RNA transport. Liang et al. created chondrocyte-targeted exosomes by fusing Lamp2b with an affinity peptide (CAP). They then used these exosomes to encapsulate CRISPR/Cas9 plasmids by fusing them with liposomes. The hybrid CAP-Exo/Cas9 sgMMP-13 was injected into rat chondrocytes to mimic osteoarthritis. Knocking out MMP-13 reduced its expression and protected cartilage from degradation. (Liang et al., 2022). Lin et al. genetically engineered exosomes by fusing HSTP1 with the exosome-enriched membrane protein Lamp2b, resulting in the creation of engineered exosomes (HSTP1-Exos). These engineered exosomes demonstrated enhanced internalization by HSC-T6 cells and a superior ability to induce the transition of hepatic stellate cells-T6 (HSC-T6) cells to a quiescent phenotype when compared to unmodified exosomes (blank exosomes) and exosomes overexpressing Lamp2b protein (Lamp2b + Exos) (Lin et al., 2022).

Moreover, due to their classification as transmembrane proteins and high levels of expression, the tetraspanin superfamily CD63/CD9/CD81 are frequently selected for genetic modification and protein fusion (Li et al., 2019). A recent study has documented the development of a new engineered exosome, which contains membrane proteins or soluble protein cargoes that are actively bound. This exosome, known as Gene-Injected Functionalized Exosomes (GIFTed-Exos), is created by incorporating the exosome-associated tetraspanin CD9 gene, resulting in the display of transmembrane proteins CD70 and glucocorticoid-induced tumor necrosis factor receptor family-related ligand (GITRL) on the exosome surface. This modification leads to the formation of GIFTed-Exos with strong T-cell co-stimulatory capabilities. The GIFTed-Exos produced exhibit significant *in vitro* and *in vivo* efficacy in the delivery of various protein cargos to specific target cells (Cheng et al., 2021). Lee et al. present a novel *in situ* labeling technique for identifying exosome cargo, which eliminates the need for exosome isolation procedures. This method involves the expression of a modified form of the engineered ascorbate peroxidase conjugated (APEX), fused with an exosome cargo protein like CD63, specifically in exosome-producing vesicles within live cells or in exosomes released into the conditioned medium. This results in the biotinylation of proteins near the APEX variant for a brief period. Mass spectrometry analysis of the biotinylated proteins in exosomes secreted by kidney proximal tu is then conducted (Lee et al., 2022).

And Kooijmann et al. conducted an experiment in which they engineered exosomes presenting GPI-anchored anti-EGFR nanobodies, resulting in a notable increase in binding affinity of these exosomes to tumor cells with varying levels of EGFR expression. Furthermore, under flow conditions, the exosomes decorated with nanobodies significantly enhanced the adhesion of cells to tumor cells that express EGFR (Kooijmans et al., 2016).

Nonetheless, altering the genes of cells that produce exosomes presents a considerable obstacle. The introduction of a targeting moiety into the exosome membrane may potentially disrupt the normal functionality of exosome membrane proteins. Furthermore, a modified purification technique must be employed to isolate specifically modified exosomes compared to the technique currently utilized for nonfunctionalized or unmodified exosomes.

#### 6.1.2 Chemical modifications

Exosomes often undergo covalent modification using techniques such as bioconjugation, amidation, aldehyde-amine condensation, and the widely employed method known as ‘click chemistry’. By utilizing these methods, the rapid formation of chemical bonds between external functional groups and bioactive compounds is facilitated (Wu et al., 2021). Smyth et al. devised a technique for attaching ligands to exosomes through the application of click chemistry. Exosomes functionalized with alkyne groups via carbodiimide chemistry were linked to a model azide, specifically azide-fluor 545. The conjugation process did not result in any significant changes in the size of exosomes, nor did it impact the ability of exosomes to adhere to or be internalized by recipient cells. These findings suggest that the reaction conditions employed had a minimal effect on the structure and function of exosomes (Smyth et al., 2014). A recent study introduced a novel nanoagent, referred to as MEXI, which was developed for the treatment of spinal cord injury (SCI) through the conjugation of bioactive IKVAV peptides onto the surface of M2 macrophage-derived exosomes using a facile and expeditious click chemistry approach. *In vitro* experiments demonstrated that MEXI effectively attenuated inflammation by modulating macrophage activity and facilitated the differentiation of neural stem cells into neurons. Furthermore, *in vivo* administration of engineered exosomes via tail vein injection resulted in targeted delivery to the site of spinal cord injury (Zeng et al., 2023a).

However, the deployment of covalent bonds in therapeutic applications poses inherent challenges, primarily attributed to their elevated stability and the requisite use of potentially toxic chemicals to induce such bonds. Hence, it is crucial to exercise caution when considering the utilization of covalent alteration techniques in therapeutic settings (Choi et al., 2021). Modifications of the exosomal membrane can occur through non-covalent methods, including binding of receptors and ligands, electrostatic interactions, and hydrophobic insertions (Nel et al., 2009; Armstrong et al., 2017). Hua et al. developed a multi-site targeting polymer with fluorescence and targeted recognition properties, capable of binding to the hydrophilic groups of CD63 through hydrogen bonding. *In vitro*, the suggested polymer demonstrated enhanced cellular uptake following its conjugation with exosomes through CD63; *in vivo*, it exhibited stable exosome binding and targeted tumor implants effectively (Hua et al., 2022). Zhu et al. collected exosomes from LECs, loaded them with doxorubicin using electroporation, and then immobilized them on IOL surfaces. *In vitro* tests showed that Dox@Exos were taken up more by LECs than free Dox, leading to better anti-proliferative effects. Animal tests showed that Dox@Exos-IOLs effectively prevented posterior capsule opacification (PCO) and were well-tolerated in the eye. (Zhu et al., 2022). Jing et al. created a nanoprobe using tumor cell exosomes (Tex) and tested its ability to image colon cancer using single-photon emission computed tomography (SPECT) and near-infrared fluorescence (NIRF). The findings confirmed the enhanced tumor-targeting ability of TEx compared to adipose stem cell-derived exosomes (AEx), highlighting the potential of exosomes in biomedical applications (Jing et al., 2021).

During genetic engineering, the combination of the targeting ligand and the exosome membrane protein gene results in the overexpression of this fusion in the recipient cells. As a result, donor cells engage in the production of genetically modified exosomes expressing the intended targeting ligands. The use of genetic engineering to modify exosomes emerges as a promising approach for incorporating functional ligands onto their membranes. Nonetheless, this approach necessitates the fabrication of plasmids and subsequent amplification of proteins in the donor cells. The exosome membrane can be targeted by both lipid and bioconjugation reactions, which have the ability to attach the targeting moiety. Chemical methods involve the bioconjugation of targeting ligands with surface proteins; nevertheless, there is a potential risk of surface protein inactivation or exosome aggregation during chemical manipulation. Despite these limitations, both strategies have demonstrated effective application (Liang et al., 2021).

### 6.2 Combined with biologically active materials for delivery

Bioactive materials exhibit the capacity to sustain prolonged exosome release and leverage their bioactive constituents for defect repair. The rapid clearance ability of the circulatory system hampers exosomes’ consistent presence at designated locations for targeted tissue repair. However, hydrogels, with favorable biocompatibility and a porous structure, serve as suitable carriers for exosomes, effectively prolonging retention in targeted regions and slowing their release (Ju et al., 2023). Zhang et al. employed hydrogels loaded with umbilical cord mesenchymal stem cell-derived exosome to fill irregular bone defects and facilitate *in situ* bone tissue regeneration in a rat femoral fracture model. The locally applied hydrogel loaded with exosome, following intramedullary nail fixation, effectively promoted fracture healing (Zhang et al., 2019). Because of the interconnected nature of bone and cartilage at joint sites, defects in both tissues may manifest concurrently, posing a challenge for single-component hydrogels to adequately address the distinct requirements of each tissue. Nikhil and colleagues created a unique bilayer gel with distinct layers mimicking bone and cartilage properties. They found that endothelial cells could help regenerate cartilage in this gel structure, suggesting it could be used for repairing osteochondral structures (A and A, 2022).

In addition to hydrogels, the utilization of bioactive scaffolds in conjunction with exosomes is anticipated. By affixing exosomes to bioactive scaffolds, not only is a favorable environment established for exosome activity, but it also addresses the limitations of the scaffold itself, including slow angiogenesis and barriers to intercellular communication. Addressing the challenge of repairing osteochondral defects in clinical practice requires understanding how biomimetic scaffolds tailored to specific microenvironments can effectively regenerate both articular cartilage and subchondral bone. A new bioinspired hydrogel scaffold made with 3D printing and decellularized extracellular matrix (dECM) and human adipose mesenchymal stem cell (MSC)-derived exosomes promotes cell attachment, migration, and differentiation *in vitro*. This approach shows potential for tissue regeneration (Li Q. et al., 2023).

In summary, bioactive materials prove to be effective carriers, preserving and safeguarding exosomes at defect sites, thereby facilitating sustained regenerative efficacy. Nevertheless, challenges persist, particularly in regulating exosome release within the scaffold. While gradual release is achievable, ensuring stable release and determining the optimal release rate remain uncertain aspects (Wang et al., 2022).

## 7 Exploration of exosomes in the diagnosis and treatment of bone degenerative diseases

Orthopaedic degenerative disorders, such as OA, OP, and IDD, pose significant challenges within the aging population. Individuals afflicted with these disorders experience pain, functional decline, and reduced exercise tolerance, leading to long-term or permanent impairments in their ability to carry out daily activities. Presently, the primary approach to managing these conditions revolves around pain relief, yet it offers limited potential for restoring function or regenerating tissue (Martel-Pelletier et al., 2016; Clouet et al., 2019). Consequently, there has been substantial interest in the utilization of exos-based therapies. In the context of OA, OP, and IDD, this section offers a brief summary of the investigations carried out on Exos, both *in vitro* and *in vivo* (Table2–5).

**TABLE 2:** Research on natural exosomes *in vitro*.

Disease	Author (year, Country)	Extraction and source of exosomes	Identification of exosomes	Cell type and source	Outcome measures	Pivotal discovery
OA	He et al. He et al. (2018) (2020 China)	BMSC(UC)	**Morphology and size**:TEM **Particle size distribution**: NTA **Protein expression**: WB	Primary chondrocytes (healthy); SD rat (ribs)	**Protein expression**: WB and IF; **Cell proliferation and migration assay:** CCK-8, transwell; **Gene quantification**: PCR	Attenuated IL-1β-induced downregulation of COL2A1 and ACAN and upregulation of MMP13 and ADAMTS5
OA	Zhang et al. Zhang et al. (2020a) (2020 China)	BMSC(UC)	**Morphology and size**:TEM **Particle size distribution**: NTA **Protein expression**: WB	RAW264.7 cell line, mouse; Primary chondrocytes (healthy); SD rat (knee joint)	**Protein expression**: WB, IF and FCM; **Cell morphology assay**:TB; **Cytokine determination**: ELISA	Macrophages treated with exosomes maintain chondrocytes’ chondrogenic characteristics
OA	Huang et al. Huang et al., (2021) (2021 China)	BMSC(UC)	**Morphology and size**:TEM **Particle size distribution**: NTA **Protein expression**: WB	Primary osteoblasts (healthy), C57BL/6J mice (knee joint)	**Protein expression**: WB; **Cytokine determination**: ELISA; **Gene quantification**: PCR and Dual luciferase reporter gene assay; **Cell cycle detection**:FCM; **Cell apoptosis detection**:FCM	BMSC-derived exosomal miR-206 promotes proliferation and differentiation of osteoblasts in OA by reducing Elf3
OA	Xia et al. Xia et al. (2021) (2021 China)	BMSC(UC)	**Morphology and size**:TEM and PhilipsMorgagni268D microscope	Primary osteoblasts (healthy), Human (purchased commercially)	**Protein expression**: WB, IF; **Cell migration assay**:transwell; **Gene quantification**: PCR, Dual luciferase reporter detection	Revealed a negative correlation within miR-125a-5p and E2F2,Chondrocytes are given an ability to endocytose miR-125a-5p
OA	Liao et al. Liao et al, (2021) (2021 China)	BMSC(UC)	**Morphology and size**:TEM **Particle size distribution**: NTA **Protein expression**: WB	Primary chondrocytes (healthy); SD rat (knee joint)	**Protein expression**: WB; **Cell proliferation assay**:CCK-8; **Gene quantification**: PCR; **Cytokine determination**: ELISA;	Suppressed inflammation, and inhibited the interleukin IL-1β-induced activation of the NF-κB pathway
OA	Li et al. Li et al. (2023a) (2023 China)	ADSC(UC)	**Morphology and size**:TEM **Protein expression**: WB	Primary chondrocytes (healthy), Human; Primary synovial fibroblasts (healthy), SD rat (knee joint)	**Protein expression**: WB,IF; **Cell proliferation assay**:EdU; **Gene quantification**: PCR, Luciferase reporter assay	MiR-376c-3p in ADSC derived Exos repressed the WNT-beta-catenin pathway by targeting WNT3 or WNT9a, and then mitigating OA
OA	Meng et al. Meng et al. (2023) (2023 China)	ADSC(UC)	**Morphology and size**:TEM **Particle size distribution**: NTA **Protein expression**: WB	Primary chondrocytes (healthy), SD rat (knee joint)	**Protein expression**: WB,IF; **Cell proliferation assay**:CCK-8; **Gene quantification**: PCR, Luciferase reporter assay; **Cell morphology assay**:TEM	MiR-429 promoted autophagy by targeting FEZ2 to ameliorate cartilage injury
IDD	Liao et al. Liao et al. (2019) (2019 China)	BMSC(UC)	**Morphology and size**:TEM **Particle size distribution**: NTA **Protein expression**: WB	Primary NPCs (healthy), Human (Intervertebral disc)	**Protein expression**: WB,IF; **Cell apoptosis detection**: TUNEL; **Gene quantification**: PCR;	MSC-exos could attenuate ER stress-induced apoptosis by activating AKT and ERK signaling
IDD	Xie et al. Xie et al, (2020) (2020 China)	MSC(UC)	**Morphology and size**:TEM **Particle size distribution**: NTA **Protein expression**: WB	Primary endplate chondrocytes (healthy), SD rat (cartilaginous endplate)	**Protein expression**: WB,IF; **Gene quantification**: PCR, RNA FISH; **Cell apoptosis detection**:FCM	MiR-31-5p negatively regulated ATF6-related ER stress and inhibited apoptosis and calcification in EPCs
IDD	Chen et al. Chen et al. (2023b) (2023 China)	ASC (Exo extraction kit)	**Morphology and size**:TEM **Particle size distribution**: NTA **Protein expression**: WB	Primary NPCs (healthy), Human (purchased commercially)	**Protein expression**: WB, IF and FCM; **Cell proliferation assay**:CCK-8; **Cell apoptosis detection**: TUNEL; **Cytokine determination**: ELISA; **Gene quantification**: PCR, miRNA sequencing; **Cell morphology assay**:ORO, ARS, AB; ROS	Moreover, miR-155-5p targeted TGFβR2 to promote autophagy and inhibit pyroptosis in NPCs
IDD	Sun et al. Sun et al. (2020) (2020 China)	NC(UC)	**Morphology and size**:TEM **Particle size distribution**: NTA **Protein expression**: WB	Primary notochordal cells (healthy), SD rat (nucleus pulposus)	**Protein expression**: WB,IF,FCM; **Cell proliferation assay**:CCK-8; **Cell proliferation assay**:EdU; **Cytokine determination**: ELISA; **Gene quantification**: PCR, miRNA Microarray Analysis	0.5 MPa/NC-exos inhibit angiogenesis via transferring high expressed miR-140-5p to endothelial cells and regulating the downstream Wnt/b-catenin pathway
OP	Li et al. Li et al. (2023b) (2023 China)	Myoblasts (UC)	**Morphology and size**:TEM **Particle size distribution**: NTA **Protein expression**: WB	C2C12 myoblasts cell line (healthy), mouse (purchased commercially)	**Protein expression**: WB,IF, FCM; **Gene quantification**: PCR, Dual luciferase reporter assay; **Cell morphology assay**: ARS	Myoblast-derived exosomal Prrx2 contributes to activating the YAP pathway, thereby facilitating the osteogenic differentiation of BMSCs
OP	Qi et al. Qi et al. (2016) (2016 China)	iPSC-MSC(UC)	**Particle size distribution**:TRPS **Protein expression**: WB	Primary BMSC (healthy), SD rat (femora and tibiae)	**Protein expression**: WB; **Gene quantification**: PCR	iPSC-MSC-Exos upregulated mRNA and protein expression of osteoblast-related genes in rBMSCs-OVX.

OA: osteoarthritis; IDD: intervertebral disc degeneration; OP: osteoporosis; BMSC: bone marrow derived mesenchymal stem cell; ADSC: Adipose-derived stem cell; MSC: mesenchymal stem cell; ASC: adipose tissue stem cells; NC: notochordal cell; iPSC-MSC: Induced pluripotent stem cell-derived mesenchymal stem cell; UC: ultracentrifugation; TEM: transmission electron microscope; NTA: nanoparticle tracking analysis; WB: western blot; TRPS: tunable resistive pulse sensing; SD, rat: Sprague-Dawley rat; IF: immunofluorescence; CCK-8: Cell counting kit-8; PCR: polymerase chain reaction; FCM: flow cytometry; TB: toluidine blue; ELISA: Enzyme-Linked Immunosorbent Assay; EdU: five-ethynyl-2-deoxyuridine; TUNEL: Triphosphate nick end-labelling; NPCs: Nucleus pulposus cells; ARS: alizarin red; ORO: Oil-red O; AB: alcian blue; IL-1β: Interleukin—1β; COL2A1: Collagen type II, alpha 1; MMP13: Matrix metallopeptidase 13; ACAN: aggrecan; ADAMTS5: A disintegrin and metalloproteinase with thrombospondin motifs 5; Elf3: E74-like factor 3; NF-κB: Nuclear factor kappa-B; ER: endoplasmic reticulum; EPC: endplate chondrocyte; Prrx2: Paired-related homeobox 2; OVX: ovariectomized; ALP: alkaline phosphatase; RNA FISH: fluorescence *in situ* hybridization; NR: not report. Bold words distinguish experimental methods.

**TABLE 3:** Research on natural exosomes *in vivo*.

Disease	Author (year, Country)	Species (age, weight, gender)	Drug delivery	Outcome measures	Pivotal discovery
OA	He et al. He et al. (2020) (2020 China)	SD rat (10 weeks, NR,male)	Intra-articular injection	**Histolog**y: H&E and SOFG Protein expression:IHC **Pain assessment**:PWT, PWL **Protein expression**: WB and IF	Significantly upregulated COL2A1 protein, downregulated MMP13 protein and the PWL value was significantly improved
OA	Zhang et al. Zhang et al. (2020b)(2020 China)	SD rat (NR, 200–250 g,male)	Intra-articular injection	**Morphology**:Micro CT **Histology**: H&E and SOFG **Protein expression**:IHC and WB **Cytokine determination**: ELISA	Exosomes alleviate cartilage damage, inhibit M1 macrophage production and promote M2 macrophage generation
OA	Huang et al. (Huang et al., 2021) (2021 China)	C57BL/6J mice (9 week and 4 week,28–24 g, NR)	Intra-articular injection	**Morphology**:Micro CT **Histology**: H&E, TUNEL, ALP and ARS **Protein expression**:WB **Cytokine determination**: ELISA	Exosomal miR-206 ameliorated inflammation
OA	Xia et al. Xia et al. (2021)(2021 China)	C57BL/6J mice (5–8 weeks,NR, male)	Intra-articular injection	**Protein expression**:WB **Gene quantification**: PCR	The facilitation of chondrocyte migration and the prevention of cartilage degeneration
OA	Liao et al. Liao et al. (2021) (2021 China)	SD rat (6 week, 250 ± 20 g,male)	Intra-articular injection	**Histology**: SOFG, TB and H&E **Protein expression**:IHC and WB	Promoted cartilage regeneration, increased chondrocyte proliferation and ECM synthesis
OA	Li et al. Li et al. (2023c)(2023 China)	SD rat (NR, 230–280 g,male)	Intra-articular injection	**Histology**: masson and SOFG **Protein expression**:IHC	Mitigated OA-induced chondrocyte degradation and synovial fibrosis
OA	Meng et al. Meng et al., 2023 (2023 China)	SD rat (4 week, 230–281 g,male)	Intra-articular injection	**Histology**: H&E and TB **Protein expression**:IHC, WB **Gene quantification**: PCR	miR-429 promoted autophagy to alleviate OA by targeting FEZ2
IDD	Liao et al. Liao et al. (2019) (2019 China)	SD rat (3 mouth, NR,NR)	Intradiscal injection	**Histology**: H&E, AB and Masson **Protein expression**:IHC, WB **Gene quantification**: PCR **Morphology**:X-ray, MRI	Delivery of MSC-exos *in vivo* modulated ER stress-related apoptosis and retarded IDD progression in a rat tail model
IDD	Xie et al. Xie et al. (2020) (2020 China)	SD rat (NR, 200–250 g,famale)	Sub-Endplate Injection	**Histology**: H&E, AB, TUNEL and SOFG **Protein expression**:IHC, WB **Morphology**: MRI	Sub-endplate injection of MSC-exosomes can ameliorate IDD
IDD	Chen et al. Chen et al. (2023b) (2023 China)	SD rat (8 week, NR,male)	Intradiscal injection	**Histology**: H&E, TUNEL and SOFG **Protein expression**:WB, FCM **Cytokine determination**: ELISA **Cell apoptosis and Reactive oxygen species assay**:FCM	*In vivo* experiments revealed that ASCs-derived exosomal miR-155-5p alleviated IDD in rats
IDD	Sun et al. Sun et al. (2020) (2020 China)	C57BL/6J mice (10–12 weeks,NR, NR)	Intradiscal injection	**Histology**: H&E **Protein expression**:IF **Morphology**: Micro CT	MPa/NC-exos were demonstrated to have a therapeutical impact on the degenerated disc with an anti-angiogenesis effect
OP	Li et al. Li et al. (2023b)(2023 China)	C57BL/6J mice (6 weeks,NR, female)	Femoral bone marrow periosteum injection	**Histology**: H&E **Protein expression**:WB **Morphology**: Micro CT **Gene quantification**: PCR	Myoblast-derived exosomal Prrx2 alleviated osteoporosis via activating the Hippo pathway
OP	Qi et al. Qi et al., 2016 (2016 China)	SD rat (12 weeks, 250–300 g,famale)	NR	**Histology**: H&E **Protein expression**:IHC **Morphology**:Micro CT	Dramatically stimulated bone regeneration and angiogenesis in critical-sized calvarial defects

OA: osteoarthritis; IDD: intervertebral disc degeneration; OP: osteoporosis; BMSC: bone marrow derived mesenchymal stem cell; ADSC: Adipose-derived stem cell; MSC: mesenchymal stem cell; ASC: adipose tissue stem cells; NC: notochordal cell; iPSC-MSC: Induced pluripotent stem cell-derived mesenchymal stem cell; SD, rat: Sprague-Dawley rat; H&E: Hematoxylin-Eosin staining; SOFG: Safranin O and Fast Green staining; IHC: immunohistochemistry; PWL: paw retraction latency; PWT: paw withdrawal threshold; WB: western blot; IF: immunofluorescence; ELISA: Enzyme-Linked Immunosorbent Assay; TB: toluidine blue; ARS: alizarin red; FCM: flow cytometry; PCR: polymerase chain reaction; AB: alcian blue; MRI: magnetic resonance imaging; TUNEL: Triphosphate nick end-labelling; ALP: alkaline phosphatase; ER: endoplasmic reticulum; Prrx2: Paired-related homeobox 2; NR: not report. Bold words distinguish experimental methods.

**TABLE 4:** Research on modified exosomes *in vitro*.

Disease	Author (year, Country)	Extraction and source of exosomes	Modification strategy (function)	Identification of exosomes	Cell type and source	Outcome measures	Pivotal discovery
OA	Xu et al. Xu et al. (2021) (2021,China)	Primary dendritic cells (UC)	E7 Peptide Surface Modification; (SF-MSC targeting capability)	**Morphology and size**:TEM; **Particle size distribution**: NTA; **Protein expression**: WB	Primary SMSCs (healthy); Human (Synovial fluid)	**Protein expression**: WB, FCM; **Gene quantification**: PCR	Targeted delivery of KGN to SMSCs leads to an increase in its effective concentration within the cell
OA	Liang et al. Liang et al. (2020) (2020,China)	Primary dendritic cells (UC)	chondrocyte affinity peptide Surface Modification; (chondrocyte targeting capability)	**Morphology and size**:TEM; **Particle size distribution**: NTA; **Protein expression**: WB	Primary chondrocytes (healthy); SD rat (knee joint)	**Protein expression**: WB; **Gene quantification**: PCR;	The cell-specific targeting property of CAP-Exo and reduced organ diffusion
OA	Wang et al. Wang et al. (2021a) (2021,China)	SMSC (NR)	miR-155-5p–overexpressing; (prevent osteoarthritis)	**Morphology and size**:TEM; **Particle size distribution**: NTA; **Protein expression**: WB	Primary chondrocytes (healthy); Human (knee joint)	**Protein expression**: WB; **Cell proliferation and migration assay:** CCK-8, transwell; **Gene quantification**: PCR **Cell apoptosis detection**:FCM	Promoted proliferation and migration, suppressed apoptosis
OA	Mao et al. Mao et al. (2021) (2021,China)	BMSC(UC)	overexpress hsa-circ_0001236, (prevent osteoarthritis)	**Morphology and size**:TEM; **Particle size distribution**: NTA; **Protein expression**: WB; **Gene quantification**: PCR	Primary chondrocytes (healthy); Human (knee joint)	**Protein expression**: WB; **Gene quantification**: PCR, Luciferase reporter assay, RNA FISH, Competing endogenous RNAs analysis	Enhanced the expression of COL2A1 and Sox9 but inhibited that of MMP13 in hMSCs induced to undergo chondrogenesis
OA	Pang et al. Pang et al. (2023) (2023,China)	BMSC (extrusion approach)	GelMA hydrogels load, (sustained release, biocompatible, excellent mechanical properties)	**Morphology and size**:TEM; **Particle size distribution**: NTA; **Protein expression**: WB	Primary chondrocytes (healthy); C57BL/6 mouse (knee joint)	**Protein expression**: WB; **Gene quantification**: PCR, **Cell morphology assay**:ORO, ARS, AB **Cell migration assay**: transwell	GelMA-NVs induce M2 macrophage polarization and inflammatory response inhibition
IDD	Luo et al. Luo et al. (2022) (2022,China)	CESC(UC)	overexpressing Sphk2 and ECM-Gels hydrogels load (prevent osteoarthritis and sustained release)	**Morphology and size**:TEM; **Particle size distribution**: NTA; **Protein expression**: WB	Primary NPCs (healthy); Rat (Intervertebral disc)	**Protein expression**: WB,IF; **Gene quantification**: PCR; **Cell apoptosis detection**:FCM; **Cell morphology assay**: SA-β-gal	Lenti-Sphk2-Exos can regulate the autophagy/senescence of NPCs and prevent disc degeneration
OP	Hu et al. Hu et al. (2021) (2021,China)	NIH-3T3(UC)	CXCR4 Surface Modification and liposome, (BMSC targeting capability and sustained release)	**Morphology and size**:TEM; **Particle size distribution**: NTA; **Protein expression**: WB	BMSC; NR	**Protein expression**: WB; **Gene quantification**: PCR; **Cell morphology assay**:ORO, ALP	Promoted osteogenesis and inhibited adipogenesis of BMSCs
OP	Zheng et al. Zhang et al. (2022) (2022,China)	PL (UC)	Conjugating with alendronate grafted PEGylated phospholipid (Bone targeting aggregation)	**Morphology and size**:TEM; **Particle size distribution**: NTA; **Protein expression**: WB	Primary BMSC and EPC (healthy); SD rat (marrow) Primary BMM (healthy); C57BL/6 mouse (marrow)	**Protein expression**: WB, IF **Cell viability assay:** CCK-8; **Cell morphology assay**:ARS, BCIP/NBT ALP Color Development; **Cell migration assay**: transwell **Cytokine determination**: ELISA	Besides directly modulating the osteogenic and angiogenic differentiation of BMSCs and EPCs,PL-exo-ALN also facilitate their coupling under GCs’ stimulation

OA: osteoarthritis; IDD: intervertebral disc degeneration; OP: osteoporosis; BMSC: bone marrow derived mesenchymal stem cell; ASC: adipose tissue stem cells; SMSC: synovial mesenchymal stem cell; CESC: cartilage endplate stem cell; UC: ultracentrifugation; TEM: transmission electron microscope; NTA: nanoparticle tracking analysis; WB: western blot; SD, rat: Sprague-Dawley rat; IF: immunofluorescence; CCK-8: Cell counting kit-8; PCR: polymerase chain reaction; FCM: flow cytometry; ARS: alizarin red; ORO: Oil-red O; AB: alcian blue; ALP: alkaline phosphatase; RNA FISH: fluorescence *in situ* hybridization; ELISA: Enzyme-Linked Immunosorbent Assay; EPC: endplate chondrocyte; ECM: extracellular matrix; COL2A1: Collagen type II, alpha 1; MMP13: Matrix metallopeptidase 13; Sox9: SRY-box, transcription factor 9; CAP: Chondrocyte-targeting; PL: platelet lysate; SA-β-gal: Senescence-associated β-galactosidase; KGN: kartogenin; GelMA: gelatin methacryloyl; NR: not report. Bold words distinguish experimental methods.

**TABLE 5:** Research on modified exosomes *in vivo*.

Disease	Author (year, Country)	Species (age, weight, gender)	Drug delivery	Outcome measures	Pivotal discovery
OA	Xu et al. Xu et al. (2021) (2021,China)	SD rat (NR,200–220 g,male)	Intra-articular injection	**Histology**: H&E, TB, and SOFG **Protein expression**:IHC	E7-exo more pronounced therapeutic effects in a rat OA model
OA	Liang et al. Liang et al. (2020) (2020,China)	SD rat (6 weeks, NR,male)	Intra-articular injection	**Histology**: H&E, TB, and SOFG **Protein expression**:IF	More prolonged cartilage localization, and deeper cartilage penetratio
OA	Wang et al. Wang et al. (2021a) (2021,China)	BALB/C mouse (NR, NR,NR)	Intra-articular injection	**Protein expression**:IHC	Effectively prevented OA in a mouse model
OA	Mao et al. Mao et al. (2021)(2021,China)	C57BL/6J mouse (8 weeks,NR, male)	Intra-articular injection	**Histology**: TB, and SOFG **Protein expression**:IHC	Intra-articular injection of exosomal circRNA_0001236 attenuated OA in the DMM mouse model
OA	Pang et al. Pang et al. (2023) (2023,China)	C57BL/6J mouse (8 weeks,20–22g, male)	Intra-articular injection	**Histology**:H&E, and SOFG; **Protein expression**:IHC,IF	GelMA-NVs effectively ameliorate osteoarthritis severity
IDD	Luo et al. Luo et al. (2022) (2022,China)	Rat (2–3 weeks,200–220 g,male)	NR	**Histology**:H&E, EdU; **Protein expression**:IHC, IF **Morphology**: MRI	ECM-Gels encapsulating Lenti-Sphk2-CESCs continuously and stably release exosomes that can pass through the AF and transport Sphk2 into NPCs
OP	Hu et al. Hu et al. (2021) (2021,China)	mouse (18 mouth,NR, male)	Tail vein injection	**Histology**:H&E; **Protein expression**:IHC,IF; **Morpholog**y:Micro-CT	The hybrid NPs specifically gathered in the bone marrow and released antagomir-188, which promoted osteogenesis and inhibited adipogenesis of BMSCs
OP	Zheng et al. Zhang et al. (2022) (2022,China)	SD rat (6 weeks, 220–250 g,male)	Intravenous injection	**Histology**:H&E; **Protein expression**:IHC,IF; **Morphology**: Micro-CT,Micro-fil perfusion	Intravenous injection of PL-exo-ALN could successfully rescue GCs induced osteoporosis

OA: osteoarthritis; IDD: intervertebral disc degeneration; OP: osteoporosis; SD, rat: Sprague-Dawley rat; H&E: Hematoxylin-Eosin staining; SOFG: Safranin O and Fast Green staining; IHC: immunohistochemistry; IF:immunofluorescence; TB:toluidine blue; EdU:5-ethynyl-2-deoxyuridine; MRI: magnetic resonance imaging; KGN: kartogenin; DMM: destabilization of medial meniscus; GelMA: gelatin methacryloyl; CESC: cartilage endplate stem cell; ECM: extracellular matrix; NP: nanoparticles; BMSC: bone marrow derived mesenchymal stem cell; GCs: Glucocorticoids; CEP: cartilage endplate; NR: not report. Bold words distinguish experimental methods.

### 7.1 Exploration of Exosomes in the clinical diagnosis and treatment of OA

#### 7.1.1 Exploration of exosomes in the clinical diagnosis of OA

As human life expectancy rises and global aging intensifies, the prevalence of OA is steadily rising. Timely diagnosis of early-stage OA is crucial for effective management and control of its progression. Nevertheless, there is currently a lack of a sensitive diagnostic modality for early OA. In clinical practice, the diagnosis of OA is typically made through a combination of imaging studies and physical examinations. However, these methods may be less reliable in detecting early-stage OA due to the subtle nature of symptoms and the lack of clear radiographic evidence, such as joint space narrowing and cartilage thinning (Crossley et al., 2008). The identification of a potential biomarker for early diagnosis in osteoarthritis has garnered interest in synovial fluid. Synovial fluid, produced by synovial membranes in joints, may contain early signs of osteoarthritis like exosomes. Studying exosomes and their contents in synovial fluid shows potential for identifying different stages of osteoarthritis (Skriner et al., 2006; Chen A. et al., 2023).

The Homeobox (Hox) gene encodes a transcription factor that plays a role in regulating limb morphogenesis and bone formation. Dysregulation of Hox has been linked to the onset and advancement of early-stage OA (Pelttari et al., 2015). Gene expression of Hox in chondrocyte-derived exosomes in synovial fluid could be a diagnostic tool for osteoarthritis. Exosomes containing miRNA can also indicate early-stage osteoarthritis. The mesenchymal stroma of the umbilical cord has high levels of miRNA140-5P, which aids cartilage regeneration (Geng et al., 2020). The impaired interaction between miRNA-140 and Let-7 has been identified as a key factor contributing to substantial growth abnormalities in chondrocytes in OA (Li et al., 2018). CircRNA_0005105 contributes to cartilage breakdown, while circ_0008365 is a potential diagnostic marker for OA found in exosomes from patient serum (Wu et al., 2017; Shuai et al., 2022). Exosomal lncRNA PCGEM1 levels were higher in people with OA, varying with disease stage, suggesting it could be used as a diagnostic marker and to distinguish between different stages of OA. (Zhao and Xu, 2018). In conclusion, the phenotypic characteristics of synovial fluid exosomes have the potential to serve as precise and efficient biomarkers for the early detection of osteoarthritis.

#### 7.1.2 Exploration of exosomes in the clinical treatment of OA

**FIGURE 3:**
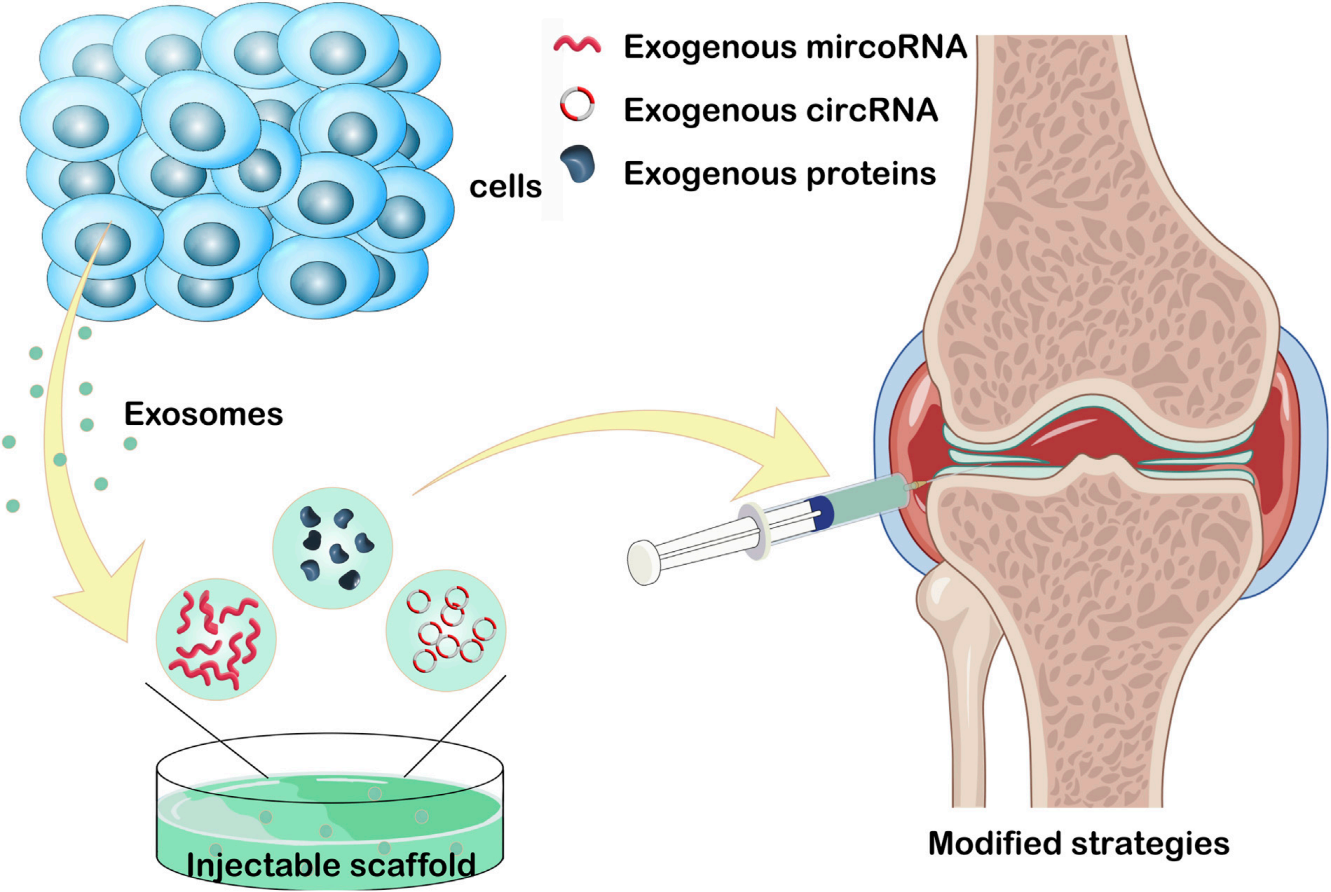
Loading exogenous circoRNA, microRNA, and proteins into exosome. Or injecting exosome into injectable hydrogels for the treatment of osteoarthritis. This figure was created using Figdraw (https://www.figdraw.com/static/index.html#/).

OA is a chronic degenerative condition significantly impacting patients’ quality of life through inflammation and progressive joint cartilage deterioration (Hu et al., 2021a; Liu et al., 2023). The etiology of OA remains elusive, posing a challenge for the development of effective therapeutic interventions (Goldring and Otero, 2011). Current treatment modalities, encompassing physical therapy and anti-inflammatory medications, focus on pain relief and disease progression deceleration. Joint replacement surgery is reserved for severe cases (Carr et al., 2012; Bowden et al., 2020). The incorporation of transcription factors within exosome encapsulation, alongside their adaptable engineering structure, holds considerable promise in anti-inflammatory therapy (Wu et al., 2018). The traditional method involves administering exosomes derived from stem cells directly to the affected region, with the goal of promoting tissue regeneration. Recently, to address constraints associated with natural exosomes, diverse modification techniques have emerged, primarily involving the integration of exogenous substances and exosome transportation through association with bioactive materials (Figure 3).

##### 7.1.2.1 Natural exosomes

Currently, a plethora of research has illustrated the significant role of exosomes in the clinical management of OA. He et al. conducted an investigation using an OA rat model, revealing that exosomes derived from bone marrow mesenchymal stem cells (BMSCs) display protective effects against cartilage damage and alleviate knee joint OA-associated pain (He et al., 2020). Zhang et al. identified that BMSC-derived exosomes promote the transition of macrophages from M1 to M2 phenotype, resulting in decreased inflammatory cytokines and increased release of anti-inflammatory cytokines. Intraarticular injection of BMSC-derived exosomes shows promising effects in reducing inflammation, mitigating cartilage damage, and impeding OA progression (Zhang et al., 2020b). Huang’s research indicates that ExosmiR-206 from BMSCs facilitates the proliferation and differentiation of osteoblasts in OA by downregulating Elf3 (Huang et al., 2021). Other studies demonstrate that BMSC-derived exosomes, rich in miR-125a-5p, effectively hinder chondrocyte deterioration in traumatic OA by specifically targeting E2F2 (Xia et al., 2021). BMSC-derived exosomes can mitigate cartilage damage in OA by inducing iron-mediated cell death through the expression of GOT1/CCR2 (Peng et al., 2023). Furthermore, ExosmiR-361-5p from hBMSCs effectively targets DDX20 and deactivates the NF-κB signaling pathway, alleviating the detrimental effects of OA (Tao et al., 2021). Exosomes from BMSCs also mitigate OA-associated inflammation through the modulation of the LYRM4-AS1/GRPR/miR-6515-5p signaling pathway (Wang et al., 2021a). Liao et al. investigated the potential of low-intensity pulsed ultrasound (LIPUS) in enhancing the therapeutic effects of BMSC-derived exosomes on cartilage regeneration in OA. LIPUS effectively enhances the regenerative properties of BMSC-derived exosomes on OA cartilage by reinforcing anti-inflammatory processes, leading to increased chondrocyte proliferation and synthesis of the cartilage matrix (Liao et al., 2021). Li et al. found that exosomes from adipose mesenchymal stem cells (ADSCs) transport microRNA-376c-3p, targeting the Wnt/β-catenin signaling axis and providing relief for OA sufferers (Li et al., 2023b). Several studies demonstrate that ExosmiR-429 from ADSC enhances cartilage repair in OA by targeting FEZ2 and autophagy mechanisms (Meng et al., 2023). Qiu et al. discovered that microRNA-129-5p in exosomes from human synovial mesenchymal stem cells (SMSCs) effectively mitigates IL-1 effects by targeting the high mobility group box 1 beta (HMGB1β) protein, ameliorating HMGB1β-induced OA symptoms (Qiu et al., 2021a).

##### 7.1.2.2 Engineered exosomes

It should be noted that pure exosomes exhibit the distinctive attribute of swift elimination within the human body, thereby imposing certain limitations on their therapeutic efficacy.

Surface engineering of exosomes has been extensively utilized in the clinical management of OA. In their research, Xu et al. reveal that engineered exosomes enable the targeted delivery of KGN to SF-MSCs, resulting in a uniform distribution of KGN within the cytosol. This targeted delivery approach increases the effective concentration of KGN within the cells and significantly enhances the chondrogenesis of SF-MSCs both *in vitro* and *in vivo* (Xu et al., 2021). Liang conducted a study on the development of chondrocyte-targeted exosomes by fusing a chondrocyte affinity peptide (CAP) at the N-terminus of the exosome surface protein Lamp2b. This fusion, combined with the liposome membrane, creates a hybrid CAP Exo capable of encapsulating the CRISPR/Cas9 plasmid. Intra-articular administration of this hybrid CAP Exo effectively suppresses matrix metalloproteinase 13 (MMP-13) expression in chondrocytes. Consequently, this intervention weakens the hydrolysis and degradation of extracellular matrix proteins in cartilage, ultimately alleviating or preventing cartilage degradation in arthritic conditions (Liang et al., 2022). Additionally, delivering miRNA-140 to chondrocytes residing within the dense nonvascular extracellular matrix of cartilage continues to pose a significant challenge. Existing scholarly sources indicate that CAP exosomes, designed as a carrier for the transport of miR-140 to chondrocytes, can selectively enter and deliver therapeutic agents to these cells. Moreover, it facilitates the delivery of miR-140 to the deep regions of cartilage via the dense medium, thereby inhibiting the activity of cartilage degradation proteases and mitigating the progression of OA (Liang et al., 2020).

##### 7.1.2.3 Gene overexpression modification

Liu et al. observed the effective mitigation of knee osteoarthritis (KOA) in a rat model through the upregulation of miR-140-5p derived from human urine-derived stem cells, which resulted in the downregulation of VEGFA (Liu et al., 2022). Wang et al. demonstrated the preventive effects against OA using exosomes derived from SMSC overexpressing miR-155-5p. This approach facilitated proliferation and migration, attenuated cell apoptosis, and regulated the secretion of the extracellular matrix in chondrocytes (Wang Z. et al., 2021). Additionally, Zhou et al. found that exosomes from synovial fibroblasts, overexpressing miR-126-3p, effectively suppressed chondrocyte inflammation and cartilage degradation in OA (Zhou et al., 2021). Moreover, studies have shown that exosomes from human SMSCs, genetically modified to overexpress miR-212-5p, effectively suppressed chondrocyte degeneration and inflammation by specifically targeting ELF3 (Zheng et al., 2022a).

In their investigation, Kong et al. discovered that ExosmicroRNA-320c derived from SMSCs facilitated the repair of cartilage damage in OA rats. This repair mechanism was achieved through the targeting of ADAM19-dependent Wnt signaling (Kong et al., 2023). The findings of Mao et al. demonstrated that the upregulation of novel ExocircRNA0001236 exosomes mitigated cartilage degradation, impeded OA progression, and augmented cartilage restoration (Mao et al., 2021). Wnt5a, identified as a factor in OA development, has been targeted by extracellular vesicles (EVs) from human mesenchymal stem cells overexpressing miR-92a-3p. These EVs promoted cartilage formation and prevented its degradation, suggesting that ExosmiR-92a-3p could potentially function as a Wnt inhibitor and serve as a therapeutic agent for ameliorating OA (Mao et al., 2018).

##### 7.1.2.4 Biomaterial binding

The potential therapeutic application of exosomes derived from MSCs in treating OA faces challenges due to limited production. However, a promising strategy has been devised in previous studies to generate high-yield exosomes with enhanced regenerative and anti-inflammatory properties, resembling MSC-derived nanovesicles (MSC-NVs). This nanovesicle induces macrophage polarization toward the M2 type and enhances the differentiation, proliferation, and migration of chondrocytes and BMSCs. Additionally, a gelatin methacryloyl hydrogel loaded with MSC-NVs (GelMA NVs) has been fabricated, exhibiting sustained release of MSC-NVs and excellent biocompatibility with favorable mechanical properties (Pang et al., 2023).

Loaded exosomes in a thermosensitive hydrogel derived from primary chondrocytes have been shown in several studies to effectively facilitate cartilage repair in individuals with OA. This occurs through the regulation of macrophage polarization, where the local release of exosomes from primary chondrocytes plays a crucial role in alleviating OA by promoting the phenotypic transformation of macrophages from M1 to M2 (Sang et al., 2022). Zeng et al. introduced a multifunctional hydrogel system inspired by mussels, enabling the simultaneous delivery and synergistic effects of MSC-derived exosomes and icariin (ICA). This mussel-inspired hydrogel, incorporating heat sensitivity, self-healing, and adhesive properties, has been successfully utilized in a joint cavity injection system, leading to enhanced cell proliferation and migration (Zeng et al., 2023b).

Moreover, an injectable hydrogel inspired by mussels, exhibiting strong adhesion properties, has been devised by certain scholars. This hydrogel facilitates the recruitment of endogenous cells and promotes the regeneration of cartilage defects (Zhang et al., 2021). It is anticipated that acellular cartilage matrix scaffolds, along with hydrogels, will act as carriers for exosomes. In a recent investigation, the introduction of an acellular cartilage extracellular matrix scaffold into cartilage defects effectively preserved administered human Wharton’s jelly–derived MSC-derived exosomes. This preservation strategy aims to enhance the healing process of rabbit osteochondral defects by harnessing the combined benefits of the acellular scaffold and exosomes (Jiang et al., 2021).

The aforementioned studies suggest that exosomes play a significant role in mitigating inflammation, restoring matrix homeostasis, and specifically addressing cartilage repair through engineering modifications. Consequently, exosomes present a promising therapeutic avenue for the treatment of OA.

### 7.2 Exploration of Exosomes in the clinical diagnosis and treatment of IDD

#### 7.2.1 Exploration of exosomes in the clinical diagnosis of IDD

Currently, the diagnosis of IDD predominantly depends on imaging techniques. Nevertheless, the onset of early IDD is often gradual, and there is a notable absence of clinical biomarkers that accurately indicate the initial stages of the disease (Wang et al., 2024b). Recently, exosomes have attracted the attention of researchers.

Sun et al. found that annulus fibrosus (AF) cells can release AF-derived exosomes (AF-exo) that are taken up by human umbilical vein endothelial cells (HUVECs). Degenerated AF-exo promoted cell migration and increased expression of inflammatory factors in HUVECs. In contrast, non-degenerated AF-exo had different effects, suggesting that AF-exo could be a potential biomarker for early IDD detection (Sun et al., 2021). Ren et al. identified the gene pairs angio-associated migratory cell protein (AAMP) and 4-aminobutyrate aminotransferase (ABAT) as potential biomarkers for IDD due to their correlation with increased CD8^+^ T cell infiltration. (Ren et al., 2023). In summary, exosomes hold promise as accurate and effective biomarkers for the early identification of IDD.7.2.2 Exploration of Exosomes in the Clinical Treatment of IDD.

#### 7.2.2 Exploration of Exosomes in the Clinical Treatment of IDD

IDD can result in various physical manifestations, encompassing, but not confined to, low back pain, dysfunction, and spinal stenosis, leading to considerable social and economic burdens. The etiology of IDD involves multiple factors, including genetic predisposition, aging, mechanical trauma, and malnutrition. Pathological alterations linked to IDD primarily entail the aging and apoptosis of nucleus pulposus cells (NPCs), progressive degeneration of the extracellular matrix (ECM), fibrosis of the AF, and an inflammatory response. Currently, conservative and surgical approaches are employed for managing IDD, contingent upon the patient’s symptoms. However, these interventions solely offer pain relief, lacking the capacity to reverse IDD or restore the mechanical functionality of the spinal structure (Xin et al., 2022).

Exosomes therapy holds significant promise as a therapeutic approach for IDD, effectively regulating metabolic processes, cellular homeostasis, and the microenvironment. This leads to the sustained release of microRNAs, proteins, and transcription factors, ultimately resulting in favorable therapeutic outcomes.

##### 7.2.2.1 Natural exosomes

A study conducted by Chen et al. unveiled the beneficial effects of BMSC-derived exosomes on IDD, attributing these effects to their antioxidant and anti-inflammatory properties. This research further affirms that targeting the NLRP3 inflammasome proves to be an effective approach for treating IDD (Xia et al., 2019). In a separate investigation, Liao et al. identified the pivotal role of BMSC-derived exosomes in regulating endoplasmic reticulum stress, preventing cell death in the nucleus pulposus, and ameliorating IDD in an *in vivo* setting. These exosomes demonstrate the capacity to alleviate cell apoptosis induced by endoplasmic reticulum stress through the activation of AKT and ERK signaling pathways (Liao et al., 2019). Evidence provided by Xie et al. indicates the protective effect of BMSC-derived exosomes on vertebral endplate chondrocytes, preventing apoptosis and calcification. This protective mechanism is primarily mediated by the miR-31-5p/ATF6 axis (Xie et al., 2020). Qian et al. demonstrated the effectiveness of exosomes derived from platelet-rich plasma in mitigating IDD by facilitating NLRP3 autophagy degradation in macrophages. Moreover, PRP-derived exosomes exhibit the capacity to alleviate IDD-associated inflammation by modulating the ubiquitination and autophagy degradation processes of NLRP3 inflammasomes (Qian et al., 2022).

**FIGURE 4:**
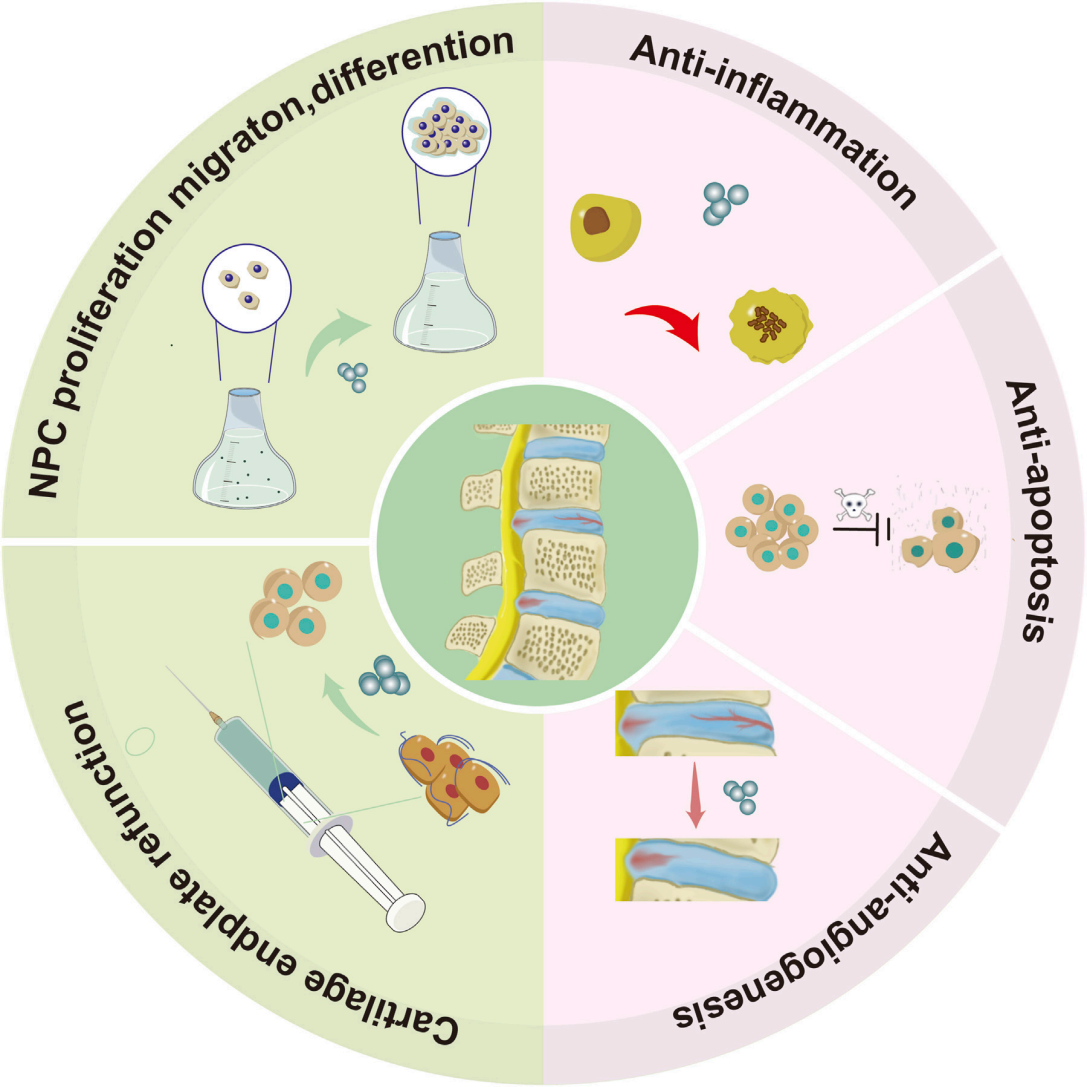
Illustrates the graphical depiction of the role played by natural exosomes in the process of IDD. These exosomes facilitate the proliferation, migration, and differentiation of NPCs, thereby aiding in the recovery of cartilage endplate function. Additionally, they exhibit anti-angiogenic, anti-inflammatory, and anti-apoptotic properties.

Concurrently, the promotion of autophagy and the reduction of pyroptosis in IDD are facilitated by human adipose tissue stem cells-derived exosomal miR-155-5p targeting TGFβR2 (Chen et al., 2023b). Sun’s research demonstrated that exosomes derived from nodal chordal cells induced by compressive load express through the miR-140-5p/Wnt/β-catenin axis, leading to the inhibition of angiogenesis and alleviation of IDD (Sun et al., 2020). Yuan et al. revealed that the introduction of exogenous miR-26a-5p via exosomes from human umbilical cord mesenchymal stem cells effectively inhibits nuclear pyroptosis through the METTL14/NLRP3 pathway (Yuan et al., 2021). Subsequent studies have unveiled the capability of exosomes from human embryonic stem cells to hinder NLRP3 inflammasomes and alleviate nuclear myeloid cell pyroptosis by transporting miR-302c (Figure 4) (Yu et al., 2023).

##### 7.2.2.2 Biomaterial binding

In the context of IDD treatment, the swift elimination of exosomes necessitates the identification of a carrier capable of sustained exosomal release within the body. As a result, the incorporation of biomaterials is employed to facilitate exosome delivery and prevent their rapid clearance. A therapeutic strategy to impede IDD was proposed by Luo et al., involving the administration of a hydrogel infused with cartilage endplate stem cells (CESCs) modified with costal cartilage extracellular matrix (ECM-Gels). Through the introduction of Sphk2 overexpression (Lenti Sphk2 CESCs) in proximity to the cartilage endplate (CEP) of rats, ECM-Gels facilitated the generation of Sphk2-engineered exosomes (Lenti Sphk2 Exos). These exosomes, expressing Sphk2, exhibited sustained release, ultimately enhancing IDD (Luo et al., 2022). Xing et al. developed a thermosensitive ECM hydrogel (dECM@Exo), which, by regulating matrix synthesis and degradation through MMPs, inhibits pyroptosis and reduces *in vitro* inflammation (Xing et al., 2021). The association between NPC aging and IDD severity is well-established. The use of MSC-derived exosomes has been demonstrated to ameliorate NPC aging and promote ECM deposition. In their study, Guan et al. devised a hydrogel incorporating exosomes based on quaternized chitosan (QCS) and oxidized starch (OST) for IDD treatment. The QCS-OST/Exos hydrogel induced senescence and rejuvenation in NP cells, stimulating ECM remodeling and partially restoring the nucleus pulposus and AF structure (Guan et al., 2023).

### 7.3 Exploration of exosomes in the clinical diagnosis and treatment of OP

#### 7.3.1 Exploration of exosomes in the clinical diagnosis of OP

Due to the multifactorial etiology of OP, it is challenging to rely on a single diagnostic component for predicting the condition. Therefore, risk score systems integrating a range of proteins, lipids, mRNA, and ncRNAs present in exosomes may serve as promising diagnostic tools for OP.

Researchers discovered abnormal tRF expression in the plasma of OP patients through small RNA sequencing on plasma exosomes. Specifically, tRF-38, tRF-25, and tRF-18 showed elevated levels in individuals with osteoporosis, suggesting they could be used as diagnostic markers for the condition (Zhang et al., 2018a). Shao and colleagues found abnormal miRNAs in exosomes of menopausal women with osteoporosis, which could be used as a biomarker for the condition (Shao et al., 2020). Chen et al. analyzed plasma exosomes from 60 patients with different bone conditions to identify new diagnostic markers. They discovered 45 proteins that were expressed differently, with four proteins confirmed. They created an exosomal protein index to distinguish between individuals with OP and those without, achieving an AUC of 0.805 for classification accuracy (Chen et al., 2020b). Hence, exosomes exhibit potential as precise and efficient biomarkers for the early detection of OP.7.3.2 Exploration of Exosomes in the Clinical Treatment of OP.

#### 7.3.2 Exploration of Exosomes in the Clinical Treatment of OP

OP, a pathological condition characterized by a gradual decline in bone mineral density, results in the degradation of bone tissue microarchitecture and an increased susceptibility to fractures. This condition is significantly associated with the aging process (Ensrud and Crandall, 2017). Postmenopausal individuals, especially those with estrogen deficiency, face a heightened risk of OP development and fractures (Eastell et al., 2016).

##### 7.3.2.1 Natural exosomes

Recent studies emphasize the substantial impact of exosomes derived from bone marrow on the bone microenvironment. These exosomes, crucial in regulating bone equilibrium, are implicated in conditions such as OP (Gao et al., 2018). Yang et al. revealed that exosomes containing MALAT1, derived from BMSCs, positively influence osteoblast activity in osteoporotic mice through the mediation of the miR-34c/SATB2 axis (Yang et al., 2019b). Additionally, exosomes RNA-150-3p from BMSCs enhances osteoblast proliferation and differentiation in individuals with OP (Qiu et al., 2021b). Myoblast-derived exosomes Prrx2 activate the Hippo pathway by transcriptionally regulating LncRNA-MIR22HG, effectively reducing OP (Li et al., 2023c). Multifunctional induced pluripotent stem cells (iPSCs) derived MSCs secrete exosomes, addressing bone defects in osteoporotic rats by enhancing angiogenesis and osteogenesis processes (Qi et al., 2016). Exosomes from BMSCs transmit miR-186 via the Hippo signaling pathway, promoting osteogenesis in ovariectomized rats (Li et al., 2021). Exosomes derived from BMSCs (STExos) enhance osteoblast differentiation of BMSCs, with BMSCs specific aptamers on the surface facilitating targeted delivery to BMSCs in the bone marrow, offering a promising treatment for OP (Luo et al., 2019).

##### 7.3.2.2 Engineered exosomes

Furthermore, the capacity of exosomes modified with C-X-C motif chemokine receptor 4 (CXCR4) to target bone tissue was discovered by Hu et al. Moreover, the introduction of antigomir-188, inhibiting bone formation and fat generation, was observed upon modifying exosomes and liposomes with CXCR4. This innovative fusion approach holds potential for OP treatment (Hu et al., 2021b). Another delivery system utilizing exosomes, derived from human iPSCs’ MSCs secretion, has been engineered to produce BT-Exo-siShn3. This modified exosome, BT-Exo-siShn3, has demonstrated the ability to inhibit osteoclast formation and promote angiogenesis, particularly in the development of H-type blood vessels. The study’s findings suggest that BT-Exo-siShn3 presents potential as a comprehensive therapeutic strategy with anti-OP effects (Cui et al., 2022).

##### 7.3.2.3 Biomaterial binding

Exosomes, derived from platelet lysate, were isolated to enhance the concentration of platelet-derived growth factors. Subsequently, these exosomes were integrated with alendronate (ALN)-grafted polyethylene glycol phospholipid (DSPE-PEG-ALN), resulting in the creation of bone-targeted PL exosomes (PL Exo ALN). This innovative formulation has shown efficacy in mitigating glucocorticoid-induced osteoporosis *in vivo* (Zheng et al., 2022b). To stimulate bone formation and facilitate the healing of defects in osteoporotic rat femoral neck canal defect models, Qayoom utilized a carrier comprising calcium sulfate/nano hydroxyapatite-based nano-cement to deliver recombinant human bone morphogenetic protein-2, zoledronate, and exosomes derived from BMSCs (Qayoom et al., 2020).

## 8 Prospects and challenges

Within the field of orthopedics, a paucity of approved therapeutic medications capable of reversing or mitigating the progression of degenerative ailments is evident. Instead, conservative strategies, such as self-management, education, physical therapy, and pain management, are commonly employed (Black and Rosen, 2016; Martel-Pelletier et al., 2016; Clouet et al., 2019). In cases where the disease advances to a more critical stage, surgical intervention emerges as the sole feasible course of action (Gamradt and Wang, 2005; Martel-Pelletier et al., 2016). Exosome technology has recently surfaced as a promising approach for managing various early degenerative ailments, extending its application beyond bone degenerative diseases to encompass nervous system degenerative diseases and retinal tissue regeneration (Zhang et al., 2018b; Rahmani et al., 2020; Deng et al., 2021; Guo et al., 2021). The diverse range of exosomal sources forms the basis for acquiring various exosomal subtypes (Yu et al., 2022). In order to enhance the concentrated presence of exosomes at the impacted area while reducing toxicity and negative consequences, different methods such as modifying engineering, increasing gene expression, and combining biomaterials are utilized to improve the effectiveness of treatment (Cheng et al., 2018; Liang et al., 2021).

However, substantial gaps and challenges persist in effectively bridging laboratory research with medical advancements. Presently, the focal point of exosome research predominantly revolves around laboratory settings, with a notable emphasis on studies conducted on small animal models. A significant impediment in the clinical translation of exosomes lies in ensuring their biosafety. Despite demonstrating favorable therapeutic effects and biosafety in cell and small animal models, the lack of comprehensive long-term *in vivo* monitoring presents substantial obstacles to successfully translating these findings into clinical applications. Therefore, in experimental settings involving primates and other sizable animals sharing microenvironments similar to humans, the evaluation of immunogenicity and toxicity can be conducted by collecting blood samples for the analysis of hematology, blood biochemistry, and immune markers, thereby laying the groundwork for prospective clinical implementations (Zhu et al., 2017; Wu et al., 2022). Furthermore, the difficulties pertaining to the extensive production and preservation of exosomes compound the barriers to clinical translation (Yamashita et al., 2018). Additionally, challenges related to the extensive production and preservation of exosomes compound the barriers to clinical translation. Currently, there is a myriad of technologies available for exosome preparation; however, the extraction procedure is intricate, associated expenses are substantial, and a lack of standardized protocols impedes the potential for commercial scalability.

## 9 Conclusion

The body of literature concerning exosomes in the realm of bone degenerative diseases is progressively growing. In the years to come, in-depth inquiries into the mechanisms through which exosomes contribute to maintaining tissue homeostasis in the skeletal system will aid in identifying the most suitable cell source for exosome production. Enhancing the biocompatibility of exosomes shows promise for boosting their therapeutic efficacy in addressing bone degenerative diseases. Furthermore, the sustained efficacy of exosomes in such conditions lacks confirmation, underscoring the need for randomized controlled trials to ascertain their true potential and safety profile.
